# Impacts of colonization on Indigenous food systems in Canada and the United States: a scoping review

**DOI:** 10.1186/s12889-023-16997-7

**Published:** 2023-10-26

**Authors:** A. Malli, H. Monteith, E. C. Hiscock, E. V. Smith, K. Fairman, T. Galloway, A. Mashford-Pringle

**Affiliations:** 1 Imperial College London, London, SW7 2AZ UK; 2 Department of Anthropology, University of Toronto Mississauga, 3359 Mississauga Rd, Mississauga, ON L5L 1C6 Canada; 3 Rehabilitation Sciences Institute, Temerty Faculty of Medicine, University of Toronto, 500 University Avenue Suite 160, Toronto, ON M5G 1V7 Canada; 4 Dalla Lana School of Public Health, Waakebiness Institute for Indigenous Health, University of Toronto, 155 College Street, 4th Floor, Toronto, ON M5T 3M7 Canada; 5 Institute for Circumpolar Health Research, 3506 MacDonald Drive, Yellowknife, NT X1A 2H1 Canada

**Keywords:** Indigenous, Food systems, Climate change, Colonization, Food sovereignty, Food security, Canada, United States

## Abstract

**Background:**

Indigenous populations in Canada and the United States (US) have maintained reciprocal relationships with nature, grounded in respect for and stewardship of the environment; however, disconnection from traditional food systems has generated a plethora of physical and mental health challenges for communities. Indigenous food sovereignty including control of lands were found to be factors contributing to these concerns. Therefore, our aim was to conduct a scoping review of the peer-reviewed literature to describe Indigenous disconnection from Indigenous food systems (IFS) in Canada and the US.

**Methods:**

Following the Preferred Reporting Items for Systematic Reviews and Meta-Analyses for Scoping Reviews (PRISMA-SR) and Joanna Briggs Institute guidelines, we searched MEDLINE, SCOPUS, International Bibliography of the Social Sciences, Sociological Abstracts, and Bibliography of Native North Americans. Data was extracted from 41 studies and a narrative review completed based on study themes.

**Results:**

The overarching theme identified in the included studies was the impact of colonization on IFS. Four sub-themes emerged as causes for Indigenous disconnection from traditional food systems, including: climate change; capitalism; legal change; and socio-cultural change. These sub-themes highlight the multiple ways in which colonization has impacted Indigenous food systems in Canada and the US and important areas for transformation.

**Conclusions:**

Efforts to reconnect Indigenous knowledge and values systems with future food systems are essential for planetary health and sustainable development. Traditional knowledge sharing must foreground authentic Indigenous inclusion within policymaking.

**Supplementary Information:**

The online version contains supplementary material available at 10.1186/s12889-023-16997-7.

## Highlights

• The main theme identified amongst the SR literature was the lasting impacts of colonization on Indigenous food systems in Canada and the US, which is described through four key areas: climate change; capitalism; legal changes; and socio-cultural changes.

• Less than 20% of included papers report author positionality, with only 7% of included papers reporting Indigenous authorship, emphasizing an opportunity for more reporting and Indigenous engagement in the future.

• Loss of cultural knowledge and practices was highlighted by many articles reviewed.

• Revitalisation of IFS must include authentic Indigenous engagement, support Indigenous knowledge frameworks, community sharing networks, education programs and co-management.

**Supplementary Information:**

The online version contains supplementary material available at 10.1186/s12889-023-16997-7.

## Background

Across the world, Indigenous Peoples^1^ have maintained considerate and intricate relationships with nature, reinforced by enduring socio-cultural beliefs that human activities should be guided by, and grounded in, respect for Mother Earth [2]. Indigenous ways of being for those living on Turtle Island (North America which is comprised of Canada, the United States and Mexico, however we have not included Mexico) have a shared understanding that we must be in relationship with the land and take care of Mother Earth, an ideology known as “kincentric ecology” [3, 4]. Upon consideration of all that Mother Earth provides, including sustenance and shelter, Indigenous Peoples hold a kinship with nature, resulting in their continued efforts to preserve the environment. This general focus that Indigenous communities place on respectful existence, and continued stewardship of nature aligns with a sustainable manner of living [2]. As such, Indigenous ecological knowledge offers a multitude of environmental advantages (i.e., increasing plant and animal populations by reducing over-harvesting; living within the limits of the surrounding environment) when compared to the colonial mindset, the dominant perspective in Canada and the United States (US), evident upon consideration that globally, Indigenous Peoples alone protect 80% of global biodiversity [5].

Beyond the environmental protection associated with Indigenous epistemologies, connections between land and health and wellbeing through the aspects of self, including the physical, emotional, spiritual and mental, are described by many Indigenous communities on Turtle Island [6]. Subsistence activities, such as hunting, fishing, and gathering, have upheld traditional Indigenous food systems for thousands of years, ensuring the components of a nutritionally adequate and sustainable diet [7]. Carried through time from generation to generation, Indigenous Peoples share acute understandings of natural ecosystems [8]. These knowledges are vast, extending from the migratory patterns of animal species and typical patterns of fruit ripening, to methods of improving biodiversity and preventing subsistence resource depletion [5, 9–11].

Traditional foods, also known as country foods^2^, are those which have been collected and eaten by Indigenous communities across many generations, with methods of harvesting and preparation that have ancestral ties [6]. These foods have been shown to promote physical health benefits for Indigenous populations by providing key nutrients and reducing the incidence of many major non-communicable diseases, such as diabetes. However, engagement in traditional food systems carries gravity far beyond physical health. The many Indigenous communities on Turtle Island have various means of engaging in traditional food systems including hunting, fishing, foraging, and agricultural activities, which are environmentally sustainable practices. Environmental sustainability is defined “as a condition of balance, resilience and interconnectedness that allows human society to satisfy its needs while neither exceeding the capacity of its supporting ecosystems to continue to regenerate the services necessary to meet those needs nor by our actions diminishing biological diversity” [12]. The intergenerational transmission of knowledge and skills required to participate in food system activities gives rise to an inherent social, and often hereditary, component to the acquisition of traditional foods [13]. Moreover, for many Indigenous communities participating in food sharing networks where surplus country foods are given to those who may be unable to provide for themselves, like Elders or seniors, is an important aspect of nutrition and culture [14]. Community feasts, collective journeying to hunting grounds, and consumption of ancestral foods are further examples of activities that contribute to the enjoyment and importance associated with traditional foods for many Indigenous Peoples [6]. Disconnection from these historical ways of existing has exacerbated mental health challenges and induced profound distress within Indigenous communities [15].

Since the introduction of colonial agricultural practices and policies, there has been increasing concern among Indigenous leaders due to the observed changes to traditional Indigenous food systems and the disregard for environmental sustainability [16, 17]. In Canada, communities have reported reduced access to country foods, alongside atypical environmental occurrences, which compromises Indigenous food security [18, 19]. Food security, and food sovereignty are significant determinants of Indigenous health and well-being [6]. Food security is defined as having “access to sufficient, safe and nutritious food that meets their dietary needs and food preferences” and “access to land and water and allows individuals to retain cultural knowledge and be culturally intact” [20, 21]. Under the United Nations Declaration of the Rights of Indigenous Peoples, Indigenous food sovereignty is defined “as the right of peoples to healthy and culturally appropriate food produced through ecologically sound and sustainable methods and their right to define their own food and agriculture systems” [22, 23], In Canada and the US, Indigenous Peoples have significantly higher rates of food insecurity when compared to their settler counterparts, often attributed to the historical and present-day impacts of colonization and systemic racism [16, 24, 25]. Disengagement with subsistence activities, including hunting, because of colonial policies has resulted in increasingly sedentary lifestyles and an inevitable push towards consumption of market foods with higher levels of sugar, salt, and additives [6].

European colonization of North America, and the subsequent establishment of settler states, required the displacement of Indigenous Peoples and the persecution of Indigenous ways of life as a fundamental element of nation-building projects [26]. In settler societies, an expectation of Indigenous communities to assimilate to the majority culture, religion and language is not only experienced, but has historically been enforced through direct state control and violence [27]. A poignant example of this imposed assimilation is the residential school system in Canada and the US, established during the 1860s [28]. This system orchestrated the ostracization of Indigenous children from their families and ancestral homelands, to enforce religious conversion and education regarding settler culture [29]. Despite the long overdue dissolution of the residential school system in 1996 and the lack of similarly overt assimilation tactics by settlers, multiple aspects of policy remain rooted in colonial ideologies [16, 30]. Legal changes over recent decades have served as barriers to traditional practices, with moratoria of subsistence activities preventing the hunting of many animal species [16]. Thousands of years of sustainable and spiritual living have consistently been placed under threat, with a lasting effect in the disruption of traditional food systems.

In recent times, Indigenous communities across the world have made strides towards regaining control over land that was seized and stolen by settlers, in attempts to achieve food sovereignty [31]. The attempts of Indigenous Peoples to revitalise and acquire jurisdiction over their food systems has shifted the focus of international research, with academics and community members examining individual food systems to determine the root causes of disconnection from traditional foods for specific Indigenous groups [32]. Moreover, investigation into potential options for reconnection, protection and revitalisation of food systems is being conducted throughout Canada and the US [33].

### Aims and objectives

This scoping review (SR) aims to identify and situate the available literature regarding disconnection of Indigenous Peoples from their traditional food systems, applying a specific focus on climate change as a potential cause, informed by a working group consisting of researchers, Indigenous Knowledge Holders, and community members. Additionally, this review will explore any stated impacts of this disconnection on Indigenous Peoples, including where solutions may exist. This review was undertaken while being mindful to ensure inclusivity of Indigenous methods of knowledge transmission. This is to make certain that the SR is representative of the communities it aims to answer questions for.

Specifically, this review aims to:

1. Scope the available literature regarding changing Indigenous food systems (IFS) in Canada and the US, focussing on climate-related changes.
2. Identify and explore causes for changing IFS in Canada and the US, including alternatives to climate-related causes.
3. Examine the impacts of disconnection from traditional food systems for Indigenous Peoples in Canada and the US.
4. Explore manners in which people try to protect IFS in Canada and the US, and the limitations of these strategies.
5. Investigate methods of food system revitalisation that have been employed for Indigenous Peoples in Canada and the US.

## Methodology

### Study design

The five following electronic databases were searched for peer-reviewed literature relating to IFS in Canada and the US: MEDLINE, SCOPUS, International Bibliography of the Social Sciences, Sociological Abstracts, and the Bibliography of Native North Americans. The search strategy focused on IFS changes or the revitalization of IFS to address the topics of interest. We only included publications published between 2016 and 2021 (the previous 5 years from when this search was conducted) as we were interested in recent work on this topic. Eligibility criteria are summarized in Table 1.

**Table 1:** Scoping review inclusion and exclusion criteria

Inclusion criteria	Exclusion criteria
Directly related to Indigenous Peoples living in Canada and the US	Not related to Indigenous Peoples or Indigenous Peoples specifically residing in Canada and the US
Related to Indigenous food systems or Indigenous food sovereignty	Not related to Indigenous food systems or food sovereignty
About Indigenous food systems changes or actions to protect or revitalize Indigenous food systems related to climate change and/or pollution	Not related to Indigenous food systems changes or actions to protect or revitalize Indigenous food systems related to climate change and/or pollution
Primary research	Not primary research (does not include data collection)
Published between 2016–2021	Not published between 2016–2021
Written in English	Not available in English
Available electronically	Papers that only document Indigenous foods (ethnobotany)
	Papers that only document the food consumption of Indigenous Peoples
	Papers that only document food literacy of Indigenous Peoples
	Papers about teaching Indigenous Peoples how to grow or cook non-Indigenous foods or utilize capitalist market-based retail environments
	Papers focused on food security or insecurity for Indigenous Peoples without discussing Indigenous food systems and experiences or changes related to climate change and/or pollution

**Fig. 1:**
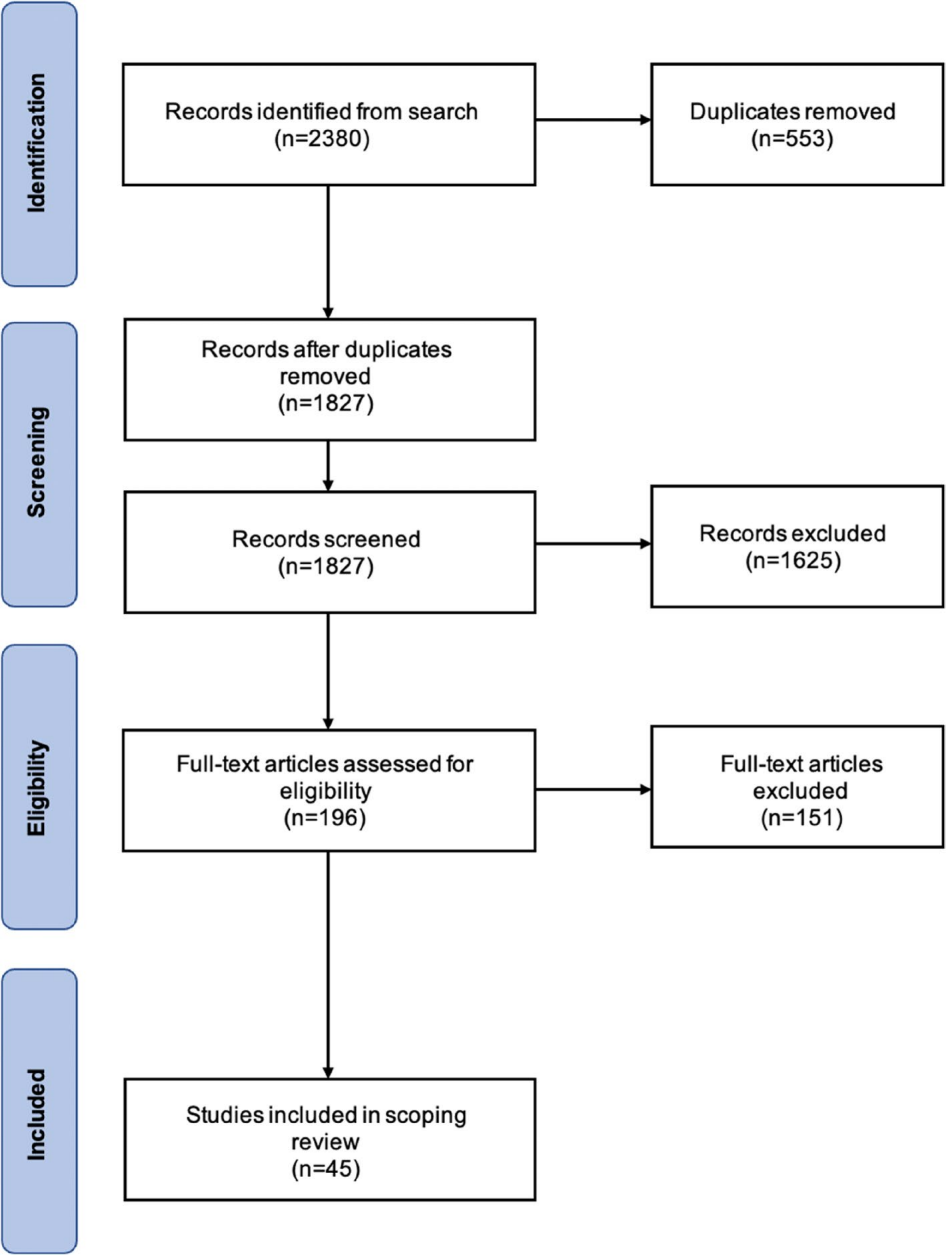
A PRISMA flowchart displaying the literature search and selection strategy employed in the scoping review

Following PRISMA guidelines for scoping reviews and the Joanna Briggs Institute, two independent reviewers screened all papers from the databases and completed full text review using Covidence Software (Melbourne, AU) [34, 35]. A detailed description of this protocol is published in the Journal of Medical Internet Research and the protocol was registered with the Open Science Framework on January 13, 2022 (Registration DOI: 10.17605/OSF.IO/XRJ87). Initially we included papers from Canada, the US, Aotearoa (New Zealand) and Australia; however, for the purpose of this review and given the similar colonial histories, climates, and Indigenous Nations in Canada and the US, we narrowed our focus during analysis to papers in these countries only. Figure 1 displays the number of records at each stage of the review process following PRISMA guidelines.

### Data review

Data were collected from the 45 included studies utilising a data extraction tool in Covidence (Table 2). The main goal of data extraction was to facilitate an overview of the included papers; however, analysis also consisted of reviewing the full-text articles in detail. Additional data regarding the ethical underpinnings of the individual study designs, including researcher positionality, was also extracted. This step was taken in a movement towards understanding the ethics behind Indigenous research, as previously published literature lacks intentional centring of Indigenous viewpoints, often taking a subconscious, colonial stance with limited inclusion of Indigenous Peoples in the design and coordination of research [36, 37].

**Table 2:**
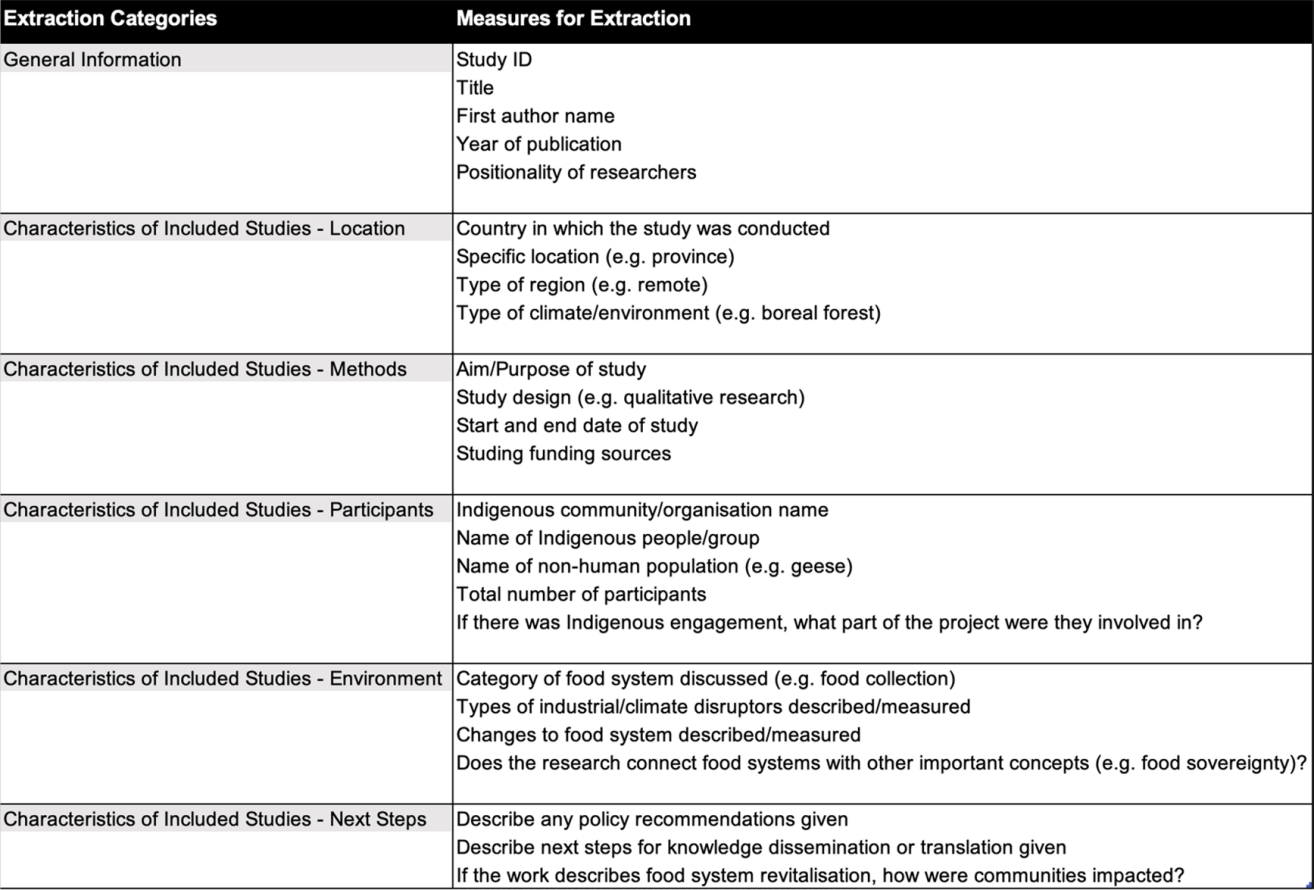
A table displaying the components of the data extraction tool used in the scoping review

We completed a review of the 41 included papers, reviewing the data extraction spreadsheet and going back to each full text article. Each full text article was analysed in detail for key concepts, which were recorded along with relevant quotes when applicable. As overlaps between the recorded concepts occurred, themes began to develop that summarized the key findings from the papers referring to the same or related concepts. Sub-themes were described if they were related to and fit within a broader theme but were worth also describing separately.

## Results

This SR includes 41 studies published between 2016 and 2021, with 34 discussing Indigenous populations in Canada and seven discussing American Indian populations in the United States. Among these, 66 different communities or organisations were discussed, including 32 distinct Indigenous groups. The group involved the most in the included papers was the Inuit (*n* = 8). Of the included studies, the majority (*n* = 20) focussed on rural communities. Thirty papers were on food collection, four on agriculture, two on the land/environment and five fell into the category of “other”, encompassing initiatives such as community education programs.

Upon reviewing the ethical grounding of each included study, eight of the 41 publications explicitly stated researcher positionality. Moreover, only three of the aforementioned eight studies included Indigenous authors. With regards to Indigenous participation, while most papers identified Indigenous participation in the research process, such as within an interview setting, it was difficult to determine the extent to which there was authentic Indigenous engagement within directing the research processes.

**Fig. 2:**
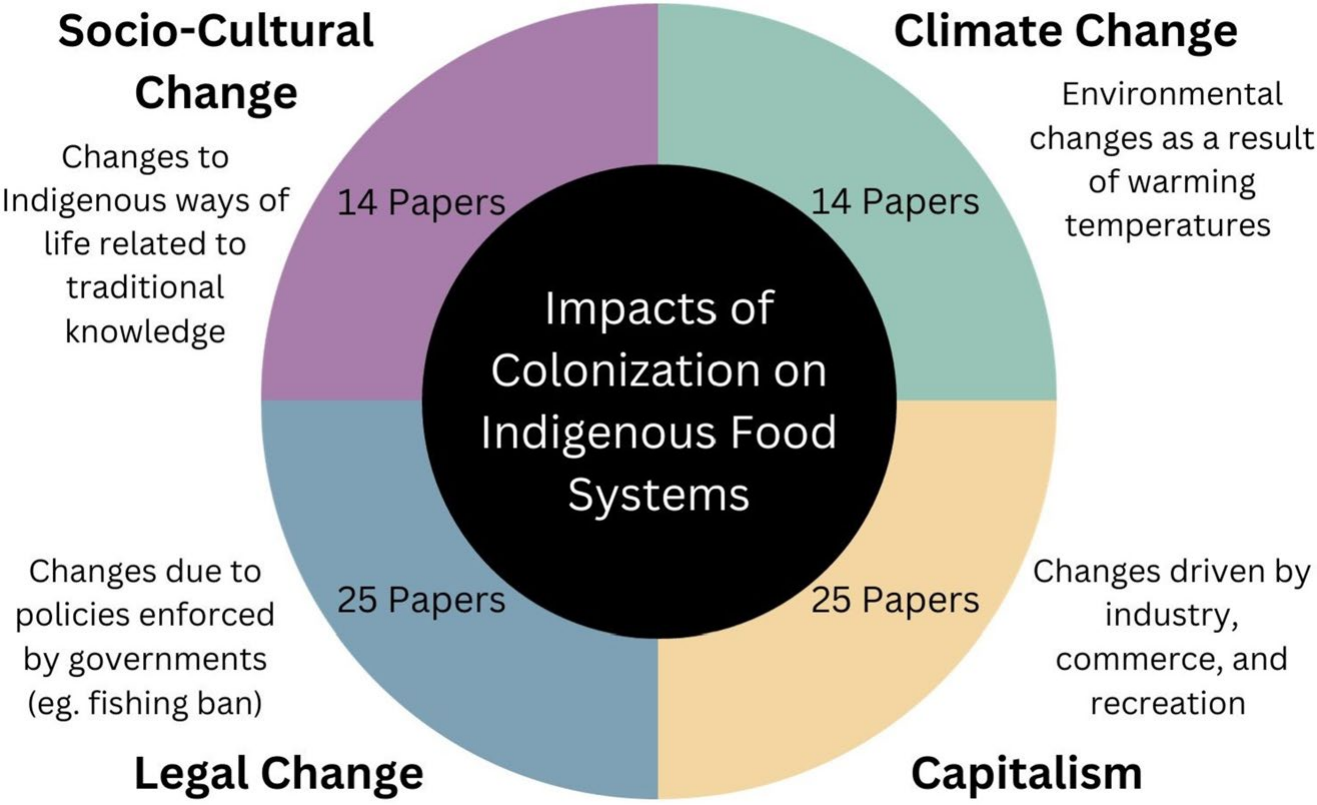
Scoping review themes and descriptions

The main theme identified amongst the SR literature was the lasting impacts of colonization on Indigenous food systems in Canada and the US. Moreover, four sub-themes that emphasize examples of the impacts of colonization on IFS were identified, including: climate change; capitalism; legal change; and socio-cultural change (Fig. 2). Whilst the literature conveys that some Indigenous communities still depend on traditional food systems for sustenance, it also discusses that others rely on a non-traditional, store-bought diet or a combination of the two, as there are numerous obstacles to obtaining preferred traditional foods [33, 38, 39]. Despite discussion of individual factors contributing to food insecurity amongst communities, each one was discussed in a capacity related to colonialism.

The main theme of the impacts of colonization on IFS is highlighted at the centre of the circle with the 4 sub-themes described in the circle quadrants. The number of papers in which each sub-theme was identified are noted in the coloured circle of the figure.

### Sub-theme 1: climate change

Of the included studies, 25 discussed climate change as a factor contributing to reduced accessibility of traditional foods for Indigenous Peoples. Traditional knowledge (TK) enables Indigenous Peoples to know which areas of the land, and times of the year, are optimal for cultivation [40]. However, this knowledge is impacted as climate change has influenced typical environmental patterns, leading to reduced country food access. For example, the ripening of bakeapples (cloudberries) has started to occur earlier than usual within harvesting season and their distribution is more geographically fragmented [40]. The vegetation growing in the palsas, the typical grounds for bakeapple growth, has changed in recent times and was attributed by the community to greater variation in temperature due to climate change.

Twenty of the 25 studies discussing climate change described a reduction in the abundance of vital animal and vegetation species within traditional Indigenous food systems, attributed to climate-related factors. Indigenous harvesters report rising sea temperatures, as well as variability in wind, fog and ice conditions as both physical barriers to being on the land, as well as reducing species abundance [38]. Beyond this, reduced harvest quality, with reported smaller size and visible signs of disease in a proportion of the land mammals and fish cultivated, have been reported by Indigenous communities, which previously occurred far less frequently. Despite Indigenous Peoples adopting modern technologies for acquisition of traditional food, such as replacing homemade hoop traps for commercial cage traps when fishing for crabs, yields continue to reduce, attributed in part to pollution [39].

### Sub-theme 2: capitalism (Industrialization, commercialization and recreational activity)

Twenty-five studies described the impacts of industrialization, commercialization, and/or recreational activity on Indigenous food systems. As aforementioned, climate change has contributed to the reduction of available country foods for Indigenous Peoples. However, many aspects of industrialization, such as the impact of shipping on marine habitats, can contribute to environmental pollution [41]. Additionally, dams and mine development on Indigenous lands can lead to habitat destruction and heavy metal pollution, reducing accessibility to species and lands traditionally utilised for food collection [42]. Infrastructure development has exerted a negative effect on species habitats, such as reducing berry abundance and quality through associated sewage disposal methods, dust from cars, and building structures, such as housing [43]. Furthermore, noise pollution from tourism has affected the migration patterns of certain species, reducing opportunities for Indigenous food collection [38].

Twelve of the studies highlighted the negative pressure that commercial and recreational harvesting activities have placed on species populations available for subsistence hunters [39, 42]. Indigenous Peoples did not fish in areas popular for commercial and recreational fishing to allow for population upsurgence [39]. However, this ultimately left less opportunity for Indigenous fishers to access country foods, reducing their engagement with traditional food systems. In addition, Indigenous and commercial fishers often seek to cultivate the same species, resulting in reduced abundance of vital, traditional species for Indigenous Peoples to access [42].

Further to this, Indigenous Peoples report challenges in confronting purported unethical behaviour of sports hunters and that tensions upon meeting these hunters on the land prevented them from going out to harvest [44]. As such, sports hunting can act as a barrier to Indigenous engagement with traditional food systems.

### Sub-theme 3: legal change

The impact of legal change, at provincial/state and governmental levels, was discussed in 14 of the included studies. In response to increasingly depleted species populations, the government began to periodically introduce moratoria within Indigenous territories [38, 40]. A 1992 ban on commercial cod-fishing within Labrador, Canada, acted as a definitive legal barrier to engaging with subsistence practices [40]. Many Indigenous Peoples made a living from working in commercial fisheries, meaning that the reduced income from sale of cod also limited the ability of communities to invest in travel to areas for traditional harvesting activities such as bakeapple picking. Similarly, a moratorium placed on the hunting of caribou, including a maximum quota that could be harvested due to waning population numbers, restricted a community in Nunavut from accessing sufficient country foods to fulfil their physical and socio-cultural needs [38]. Fishing activity in Saugeen, Canada, was significantly limited by the government in the early 1900s, leading to a reduction in engagement with country foods [45].

Indigenous Peoples have reported frustration towards provincial governments due to a lack of regulation contributing to unethical behaviour amongst sports hunters [44]. Within Indigenous communities, internal agreements prohibit animal harvesting during important life cycle stages, such as infancy or fertile periods, considered especially important in the face of dwindling populations. However, communities in the Peace River Region of Canada have noted significant sports hunting of cow moose, despite reduced species numbers. As such, Indigenous communities do not harvest these animals in order to preserve future populations.

Despite Indigenous communities feeling gratitude for land legislation protecting traditional territories, it has made it more challenging to access these areas to engage in subsistence activities [46]. Inuit populations across Inuit Nunangat (Nunavut, Northern Quebec, Labrador, and Northwest Territories) felt that governmental wildlife management was inconducive to sustainable living and could lead to complete resource depletion of caribou [16].

### Sub-theme 4: socio-cultural change

Socio-cultural change amongst Indigenous populations was a focus within 14 of the included studies but was discussed in smaller instances across 26 of the SR publications. A decline in food-related traditional behaviours was reported, including a reduction in sharing practices within an Ojibway community in Ontario, Canada, compared to historical norms [45].

Twelve of the included studies conveyed that younger generations of Indigenous Peoples have grown increasingly disconnected from traditional foods, attributed to a disruption of intergenerational knowledge transmission which can lead to a reliance on store-bought foods and, consequently, changing food preferences [42, 47]. There are fewer opportunities for youth education and participation in subsistence activities, due to the trickle-down effect of adult disconnection from these activities due to climate change and sports hunting [44]. Moreover, it has been suggested that, at times, younger generations also appear less engaged in harvesting [40]. Limited cultivating skills amongst Indigenous Peoples have been discussed as a cause for disengagement with traditional food systems, an expected outcome of generations of “cultural genocide” that intended to break the chain of intergenerational knowledge transfer and the ability of Indigenous Peoples to connect to the land [42]. As the quintessential tool of cultural genocide, residential schools for Indigenous Peoples have led to lasting socio-cultural impacts, causing a shift from subsistence to cash economies [48]. As such, Indigenous adults and youth gravitate towards market-foods over traditional foods, due to enforced assimilation.

Shifting economic pressures and alternative employment were discussed as a barrier to consumption of traditional foods. Due to consistent reduction in abundance of muskrat populations for harvesting, a sustainable income from selling muskrat fur was not attainable for Gwich’in and Inuvialuit communities in the Northwest Territories, Canada [49]. As a result, hunting muskrats transitioned culturally, from a manner of making a living to an occasional activity. The consumption of less muskrat meat is a repercussion of this, further disconnecting Indigenous Peoples from country foods.

## Discussion

The purpose of this SR was to explore the peer-reviewed literature published from 2016 to 2021 to understand changes to Indigenous food systems, such as climate change, and the subsequent impacts on Indigenous communities in Canada and the US. Additionally, this SR looked to determine how Indigenous Peoples are protecting and revitalising their ancestral, food-related traditions to determine optimal practices and areas for further study. Our SR methods were framed with the goal of combatting the perpetuation of a Eurocentric perspective within this review.

Despite slight variation in traditional food abundance being described as “normal” within the included literature, it is evident that IFS changes, categorized as four sub-themes, have resulted in Indigenous Peoples experiencing incredible challenges when attempting to engage with traditional food systems [40]. Our findings are supported in the wider literature suggesting that climate change has led to limited traditional food availability for Indigenous Peoples in Canada and the US [18]. However, we advance this narrative by describing interaction of climate change with three other factors, initially and continually perpetuated by colonialism, that have led to disconnection from food systems. European colonization introduced significant industrialization to Canada and the US, with inevitable ramifications of increased pollution and habitat destruction contributing to climate change [42, 50]. Furthermore, the industrialization and forced assimilation as part of colonization triggered a tremendous socio-cultural shift away from tradition for Indigenous Peoples [50].

Traditional foods and the associated activities required to acquire them, such as community agriculture and hunting, contribute to the shared epistemologies of Indigenous Peoples in North America, in part related to the intricate ties between traditional foods and creation stories [3, 6]. Within these retellings, the moral grounding lies in respect for the Earth and the species it provides [3, 51]. As such, it becomes clear how engagement in traditional food activities can constitute spiritual practice for many Indigenous Peoples [43].

Additionally, throughout the SR literature, a depiction of traditional foods as more nutritionally valuable than store-bought alternatives, alongside genuine enjoyment associated with their cultivation and consumption, was apparent [49]. Traditional food is considered more nutritious when compared to market food [33]. Given the limited access to the diets that have sustained Indigenous Peoples for thousands of years, the “nutrition transition”, a phenomenon which describes the movement away from traditional food consumption to market and processed foods,, has contributed to unprecedented rates of non-communicable diseases amongst Indigenous communities [52, 53]. Inability to obtain fresh country foods is causing an over-reliance on market foods, as well as subsequent shifts in food preferences amongst Indigenous Peoples, conditions detrimental to health [42]. Furthermore, the medicinal value of traditional foods is ingrained within Indigenous epistemology [47]. Several studies within this SR discussed Indigenous reliance on traditional foods for medicine, in opposition to colonial methods of treating illness [43, 47]. Disconnection from traditional food systems, therefore, is contributing to new health issues for Indigenous Peoples, whilst removing their capacity to treat them.

Engagement in subsistence activities is inherently social for Indigenous Peoples, as they learn from, and cultivate with, community members [40]. Journeying to hunting grounds, sharing harvested foods, and participating in ancestral practices have demonstrated mental health benefits for communities [49]. This is corroborated by description of being on the land and maintaining connections to nature as “therapeutic”, where these connections are vital for Indigenous Peoples as evidenced by the negative health, social, and economic outcomes directly related to displacement from lands. The aspects being described by many Indigenous Peoples, including physical, emotional, spiritual and mental, are evidently and reciprocally dependent upon traditional food systems [6]. Additionally, the changing food landscape as a result of the nutrition transitionand the emerging dependence on cash economies has impacted wellbeing by removing traditional sources of income, such as the sale of muskrat, and creating need for employment outside the traditional realms of being [49]. This has further implications for intergenerational transmission of knowledge as time-restrictive work, coinciding with shifts towards individualistic behaviour for adults, reduces opportunities for younger Indigenous Peoples to witness, and engage in, food-related practices [40, 42].

Given the diverse impacts of changing traditional food systems, the studies reviewed propose a myriad of solutions addressing both the tangible, physical food access issues, and continuing cultural disconnection. Spiegel et al. describe that, for Indigenous Peoples, witnessing the shifting landscapes of their homelands can contribute to “solastalgia”, defined as the difficulties that people with strong ties to their home environment can experience upon witnessing its deterioration [41]. Given that Indigenous Peoples have historically maintained an intimate relationship with the Earth, difficulty witnessing environmental degradation is unsurprising [2]. However, this relationship between Indigenous Peoples and their homelands drives their collective willingness to engage in food system revitalisation strategies [54].

Upon discussion of protection and revitalisation of Indigenous food systems in the face of climate change, a variety of solutions are offered within the SR literature (Table 3). Anderson et al. asserts that the “adaptive capacity” of individual communities, defined as the ability to adapt to and combat external food system disruptors, is underpinned by a myriad of inherent strengths present in Indigenous communities [40]. Firstly, the socio-cultural significance of country foods, and the subsequent enjoyment garnered from cultivation processes, contributes to adaptive capacity by ensuring that Indigenous Peoples continue to seek opportunities to attain them, even in the face of adversity, including climate change. Other Indigenous adaptive capacity attributes include TK and sharing networks, as TK helps communities uncover patterns in environmental changes to aid cultivation, whilst sharing networks ensure that people with limited access can still engage with traditional foods [14]. Modern technology, such as speedboats to reach more distant hunting grounds, can also enhance adaptive capacity, but typically carry the disadvantages of high financial cost and lead to long-distance travelling, which can be challenging for Elders [40].

**Table 3:** Considerations for indigenous food systems protection, adaptation, and revitalization

Indigenous Communities	Traditional Knowledge
	Community Sharing Networks
	Technology
Partnerships	Co-management between Indigenous groups and government or industry
	Education Programs
	Indigenous Leadership

Implementation of country food markets was also suggested in response to reduced traditional food access [32, 55]. Following successful implementation in Greenland, Ford et al. describe the benefits associated with markets in which Indigenous Peoples can purchase country foods [55]. Not only could they provide a social gathering space, but communities experiencing difficulties with harvesting traditional foods would still be able to obtain their associated nutritional benefits. However, communities remained sceptical towards market introduction, due to the potentiality of resource diversion away from sharing networks, which often serve populations potentially unable to provide for themselves, including the elderly [14, 55]. Compromising these networks would serve to undermine Indigenous belief systems by contradicting core principles of community sharing [55]. Additionally, commodifying country foods could introduce unethical behaviour to Indigenous food systems, with financial rewards introducing an incentive for resource depletion. Moreover, country food markets provide no resolution for specific socio-cultural issues resulting from traditional food systems fragmentation, such as poor mental health from not partaking in subsistence activities on the land [15].

Co-management between Indigenous Peoples and the government was proposed widely in the reviewed literature. Despite centuries of colonial prejudice towards Indigenous ways of knowing, community TK and management systems carry inherent benefits of proven sustainability [47]. As described by Berkes, “both science and Indigenous knowledge are “legitimate in [their] own right, within [their] own context; each has its own strengths…in parallel enriching one another as needed” [56]. However, throughout the literature, a dichotomy was evident between the ethics underpinning industry and those underpinning Indigenous conduct. TK guides reciprocal, sustainable environmental practice, conveyed by Ban et al. upon explaining Indigenous halting of subsistence activities to allow depleted populations time to recover [39]. This was corroborated by Gilbert et al. highlighting Indigenous leaders encouraging communities to “stay patient with nature” when experiencing low-yield harvests [38]. Co-management with industries or governments would allow for Indigenous self-advocacy and influence on policy. However, Cruickshank et al. explain that co-management remains dependent upon information, power, and trust sharing between Indigenous communities and their collaborators [46]. This process may prove challenging due to Indigenous hesitancy surrounding sharing TK due to fear of culturally inappropriate knowledge use and the historically extractive colonial practices of governments and industries.

Additionally, educational programs and workshops, exploring topics such as farming and traditional food preparation, could encourage food system revitalisation [48, 57]. One example is the “Community Champions” model, which empowers Indigenous Peoples to deliver workshops on food preservation to their peers [57]. Whilst being a step towards food revitalisation, the workshops also promote community connection. Moreover, Delormier et al. highlight the importance of youth involvement in these programs to ensure the sustainability of traditional Indigenous practices, through improving intergenerational transmission of knowledge [58]. However, Tsuji et al. highlight that co-ordinating these programs can require financial investment, potentially limiting their feasibility [59].

Beyond food systems, limited discussion of researcher positionality in the reviewed studies serves to potentially undermine their validity, due to difficulties determining the engagement levels with, and authentic understanding of, Indigenous Peoples. This is highlighted by messaging in the literature which appears to contradict Indigenous ways of knowing, such as alluding to the elderly as “vulnerable” [55]. Ensuring Indigenous engagement in research processes can ensure the authenticity of narratives conveyed [37]. This is critical, especially upon considering that Indigenous Peoples are calling for further research into traditional food systems [38].

As Indigenous Peoples embark on the journey towards reclaiming their food systems and therefore ensuring access and control of lands, achieving food security and sovereignty becomes increasingly feasible. This will serve to equalise processes such as co-management, as Indigenous Peoples can share their TK whilst maintaining country food access. It is imperative that industries and governments understand that the improved sustainability of extractive practices, and the ability to combat climate change, will require collaboration with Indigenous populations [46]. By bridging the gap between Indigenous ways of knowing and colonial mindsets, we stand to improve industrial ethics and venture towards sustainable cultivation, embracing the relationships of respect with the land that have been practiced and safeguarded by Indigenous Peoples since time immemorial [16, 31].

### Limitations

Given the complex interplay of factors contributing to IFS changes, the SR search strategy may have been too specific, resulting in the potential to miss relevant literature. Therefore, some studies discussing alternative causative factors other than climate change may have been excluded. Moreover, relevant Indigenous-led research may have been missed as this work is underrepresented in the peer-reviewed academic literature and we excluded papers not written in English due to feasibility and Indigenous Peoples living on Turtle Island speak and write in a variety of languages. Additionally, the exclusion of two papers from Australia and Aotearoa (New Zealand) limits the international generalisability of the SR findings. Our time restriction to literature published between 2016 and 2021 may have also excluded important papers for this review despite our goal to only consider recent work. 

## Conclusion

This review highlights colonization in Canada and the US as the premier cause for Indigenous disconnection from land and therefore traditional food systems, through a complex interplay of factors including climate change. The resulting impact involves a host of physical, mental, and social consequences for Indigenous Peoples, who are increasingly unable to partake in their traditional food systems practices. In continued efforts to achieve food security and sovereignty for communities, consideration of revitalisation techniques, including education programs and co-management, is imperative. Ongoing work on this topic must include authentic Indigenous engagement and respectful handling of TK to ensure culturally appropriate management of traditional practices.

### Supplementary Information

**Additional file 1: Table 1. **A table displaying the included studies within the scoping review and their respective characteristics.

## Data Availability

Additional information regarding our search strategy is available upon request and details outlined in our published protocol (https://www.researchprotocols.org/2023/1/e41627).

## Abbreviations

IFS: Indigenous Food Systems
SR: Scoping Review
TK: Traditional Knowledge
US: United States

## Footnotes

1 Within this paper, we define Indigenous Peoples according to the José R. Martínez Cobo Study working definition: *Indigenous communities, peoples and nations are those which, having a historical continuity with pre-invasion and pre-colonial societies that developed on their territories, consider themselves distinct from other sectors of the societies now prevailing on those territories, or parts of them. They form at present non-dominant sectors of society and are determined to preserve, develop and transmit to future generations their ancestral territories, and their ethnic identity, as the basis of their continued existence as peoples, in accordance with their own cultural patterns, social institutions and legal system* [1].

2 Traditional foods and country foods will be used interchangeably throughout this paper.
