# Supplementary material for: Impacts of colonization on Indigenous food systems in Canada and the United States: a scoping review
Source: BMC Public Health. 2023 Oct 26;23:2105. doi: 10.1186/s12889-023-16997-7 (PMC10601184; doi:10.1186/s12889-023-16997-7)
Supplement: Supplementary file 1 — Additional file 1: Table 1. A table displaying the included studies within the scoping review and their respective characteristics. [file 12889_2023_16997_MOESM1_ESM.docx]

**Supplementary File**

**Table 1:** A table displaying the included studies within the scoping review and their respective characteristics.

*
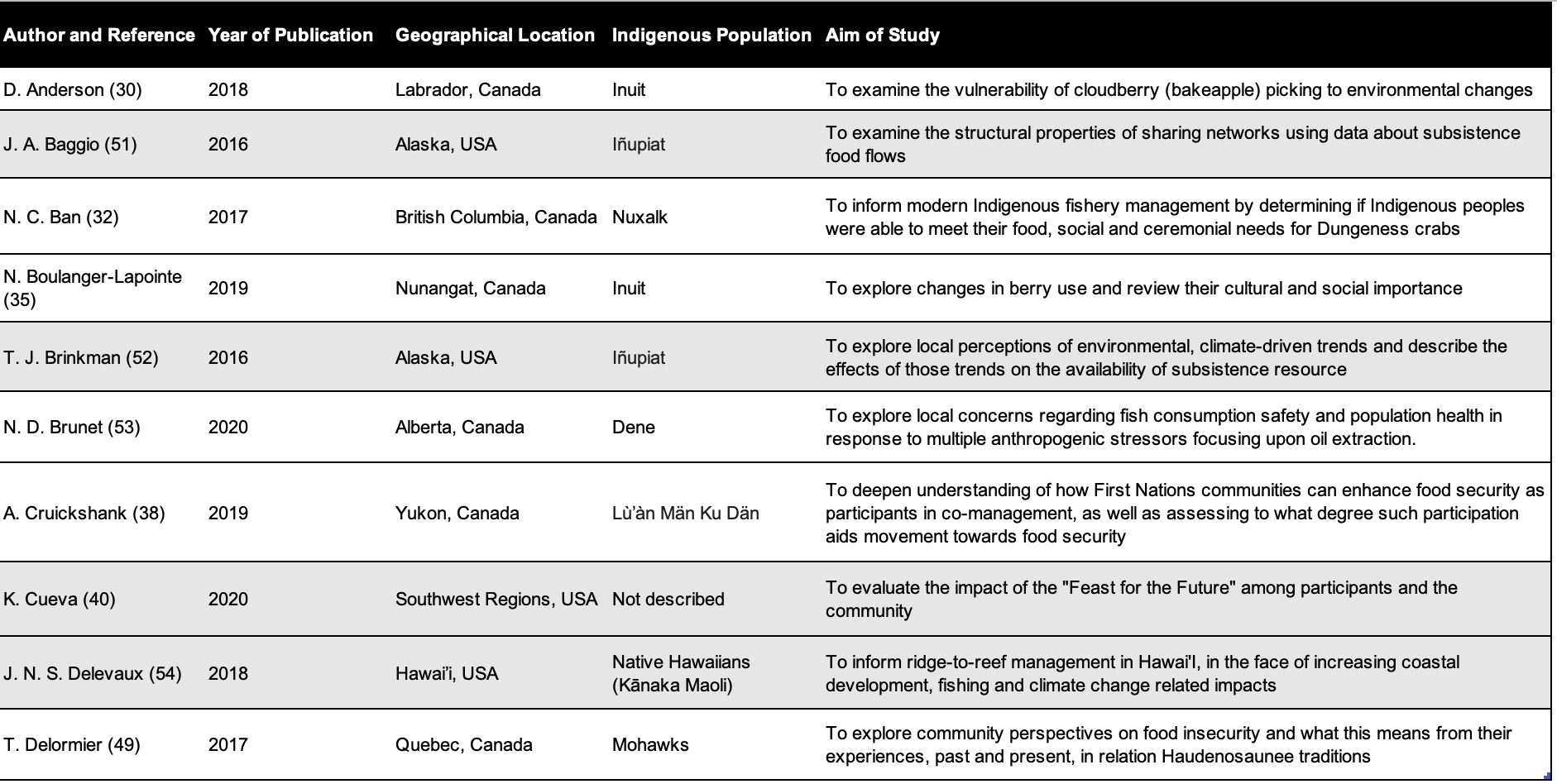
*

*
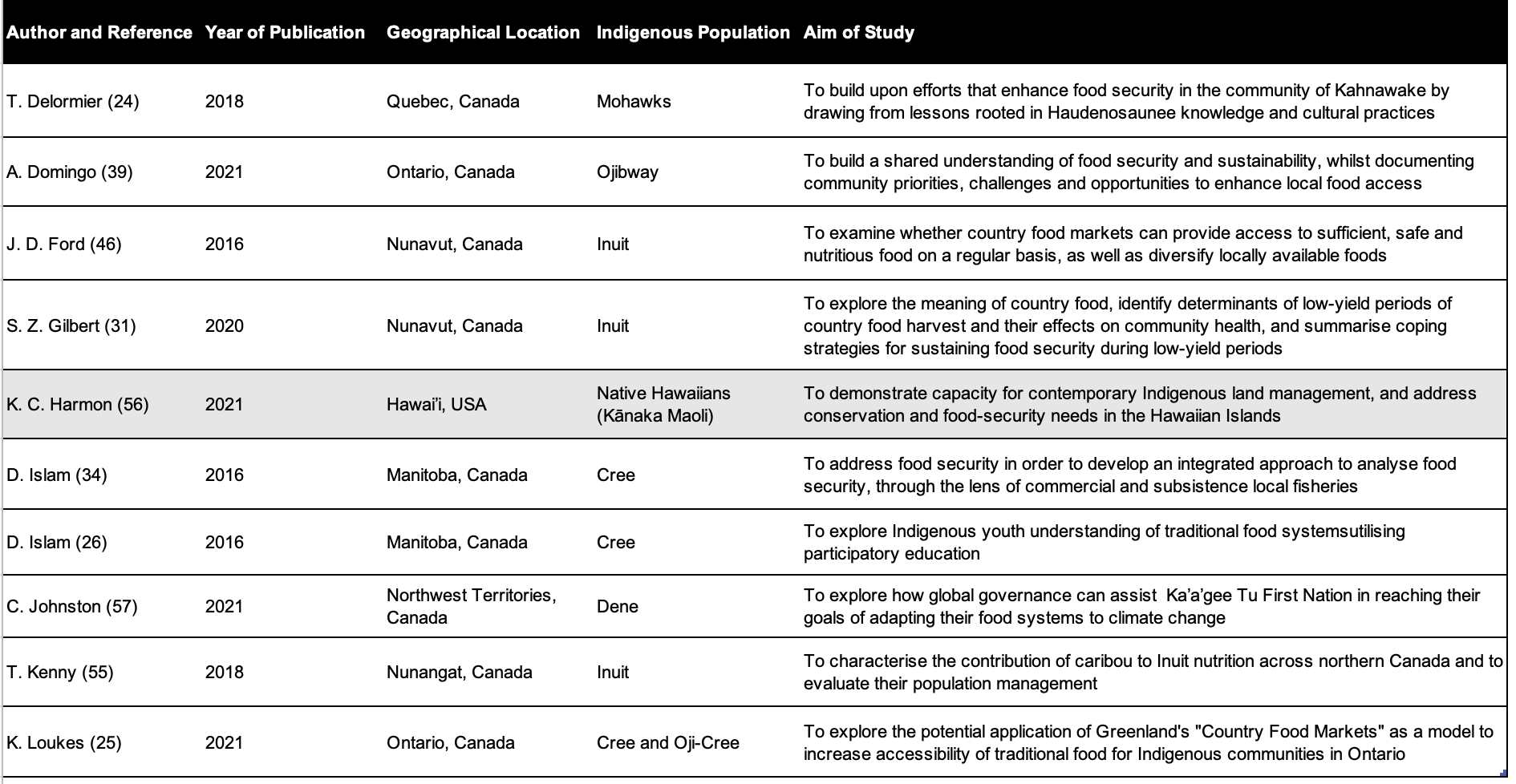
*

**Table 1:** Continued


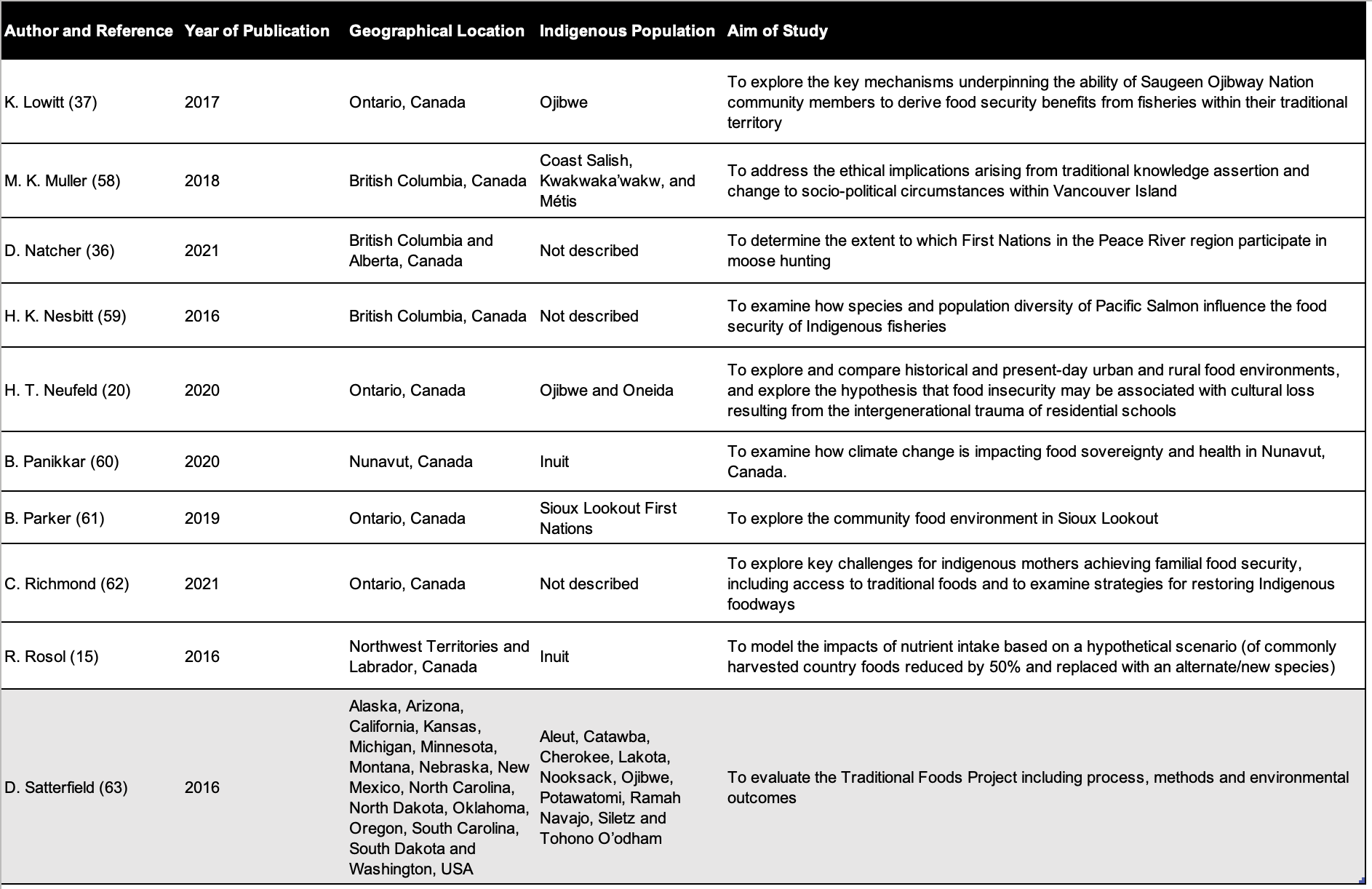


*
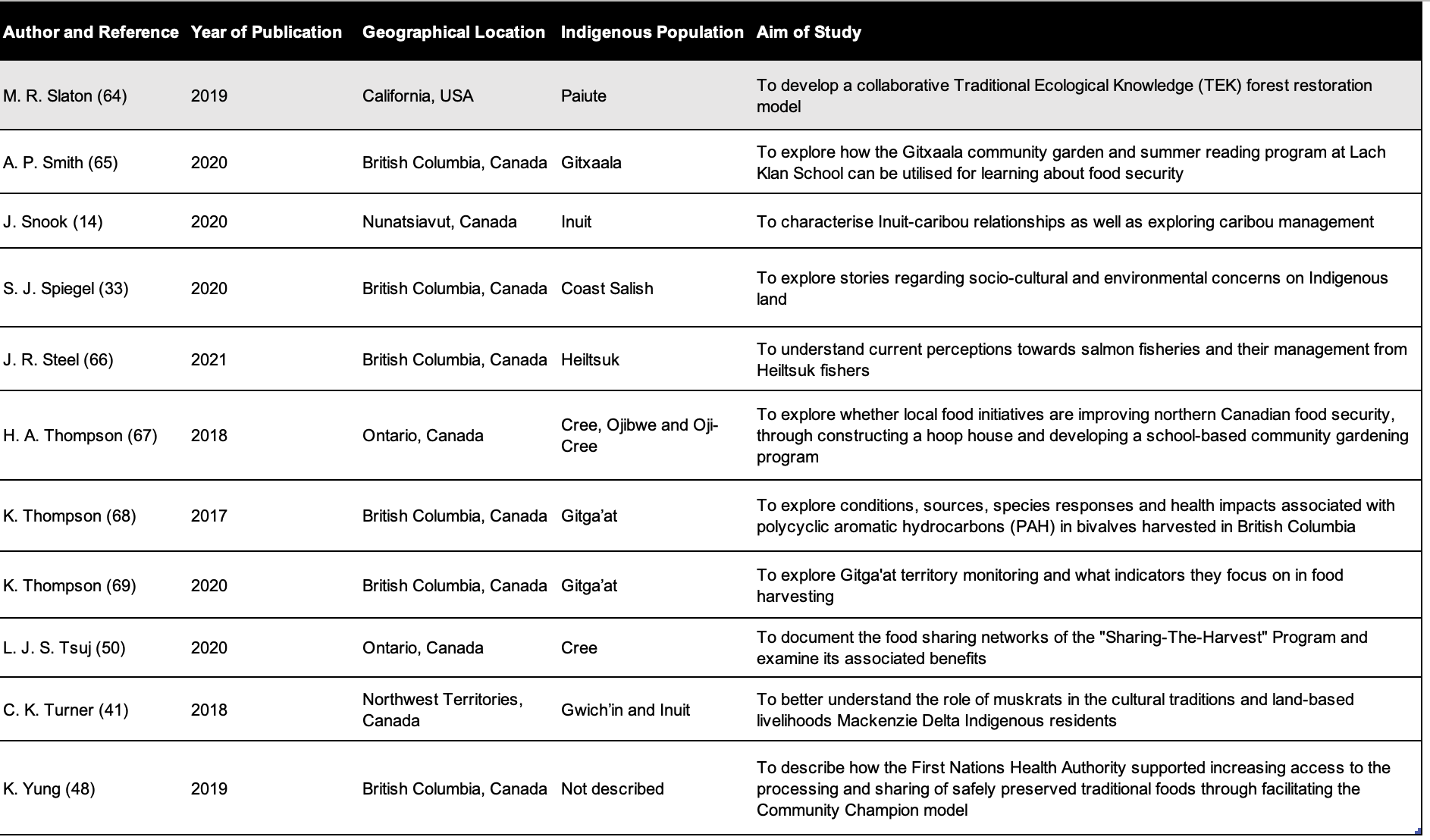
*
